# Effectiveness of end-stage renal disease communication skills training for healthcare personnel: a single-center, single-blind, randomized study

**DOI:** 10.1186/s12909-022-03458-9

**Published:** 2022-05-23

**Authors:** Ji-Tseng Fang, Shih-Ying Chen, Ya-Chung Tian, Chien-Hung Lee, I-Wen Wu, Chen-Yi Kao, Chung-Chih Lin, Woung-Ru Tang

**Affiliations:** 1 Kidney Research Center, Department of Nephrology, Linkou Chang Gung Memorial Hospital, Taoyuan, Taiwan; 2 School of Nursing, College of Medicine, Chang Gung University, 259 Wen-Hwa 1st Road, Gueishan Dist, Taoyuan, Taiwan; 3 Department of Nephrology, Keelung Chang Gung Memorial Hospital, Keelung, Taiwan; 4 Division of Hematology-Oncology, Department of Internal Medicine, Taoyuan Chang Gung Memorial Hospital, Taoyuan, Taiwan; 5 Department of Computer Science and Information Engineering, Chang Gung University, Taoyuan, Taiwan

**Keywords:** Communication, Online education, Nephrology, Continuing medical education, Shared decision-making, Truth disclosure

## Abstract

**Background:**

Given that the consequences of treatment decisions for end-stage renal disease (ESRD) patients are long-term and significant, good communication skills are indispensable for health care personnel (HCP) working in nephrology. However, HCP have busy schedules that make participation in face-to-face courses difficult. Thus, online curricula are a rising trend in medical education. This study aims to examine the effectiveness of online ESRD communication skills training (CST) concerning the truth-telling confidence and shared decision-making (SDM) ability of HCP.

**Methods:**

For this single-center, single-blind study, 91 participants (nephrologists and nephrology nurses) were randomly assigned to two groups, the intervention group (IG) (*n* = 45) or the control group (CG) (*n* = 46), with the IG participating in ESRD CST and the CG receiving regular in-service training. Truth-telling confidence and SDM ability were measured before (T0), 2 weeks after (T1), and 4 weeks after (T2) the intervention. Group differences over the study period were analyzed by generalized estimating equations*.*

**Results:**

IG participants exhibited significantly higher truth-telling confidence at T1 than did CG participants (t = 2.833, *P =* .006, Cohen’s d = 0.59), while there were no significant intergroup differences in the confidence levels of participants in the two groups at T0 and T2. Concerning SDM ability, there were no significant intergroup differences at any of the three time points. However, IG participants had high levels of satisfaction (*n* = 43, 95%) and were willing to recommend ESRD CST to others (*n* = 41, 91.1%).

**Conclusions:**

ESRD CST enhanced short-term truth-telling confidence, though it is unclear whether this was due to CST content or the online delivery. However, during pandemics, when face-to-face training is unsuitable, online CST is an indispensable tool. Future CST intervention studies should carefully design interactive modules and control for method of instruction.

**Supplementary Information:**

The online version contains supplementary material available at 10.1186/s12909-022-03458-9.

## Background

According to the United States Renal Data System, the prevalence of end-stage renal disease (ESRD) in Taiwan has been the highest worldwide since 2001 [1], which places a substantial financial burden on Taiwanese public health insurance [2]. ESRD causes 1 billion US dollars in medical expenses each year, which is equal to approximately 50% of total expenses paid by public health insurance for outpatients suffering from major illnesses. Furthermore, ESRD is often complicated by other chronic diseases, such as hypertension, diabetes mellitus, and cardiovascular issues. As multiple undesirable outcomes are associated with ESRD, communication with patients becomes a crucial issue in this context.

Because 85% of medical-related lawsuits in the US and numerous patient safety reports are caused by miscommunications [3], as shown in the annual report of the Taiwan Patient Safety Reporting System, we believe that “communication” should be the core component of collaborative practices. The most common and challenging issues concerning clinical communication are related to “truth-telling” and “shared decision-making (SDM)” [4]. Truth-telling usually entails conveying bad news, which refers to any message that can lead to serious consequences [5]. The SHARE model in Japan is one of the most well-known international theoretical frameworks associated with truth-telling and is suitable for use in Eastern countries. The SHARE model consists of four components: S, setting up a supportive environment; H, considering how to deliver bad news; A, discussing additional information; and RE, providing reassurance and emotional support. The model particularly emphasizes the RE component because that component is most important for patients [6]. In addition to truth-telling, health care personnel (HCP) also need to discuss treatment options with patients. SDM is “an approach where doctors and patients share the best available evidence when faced with the task of making decisions, and where patients are supported to consider options, to achieve informed preferences” [7]. Dr. Elwyn proposed the three-talk model, the most concrete theoretical framework for SDM, which is extensively used in different countries [7, 8].

Unfortunately, curricula addressing these aspects are not emphasized either in higher education or in-service training [9, 10]. Thus, this study designed a communication skills training (CST) program focused on truth-telling and SDM. In particular, given the fact that the treatment decisions made for ESRD patients are long-term and significant, patients need to be able to discuss their treatment options and preferences with the medical team. After fully understanding the advantages and disadvantages of all potential treatment options, patients can make informed decisions based on their treatment preferences. This process of decision-making is the essence of SDM [7, 11].

In the past, CST programs in Taiwan have predominantly been conducted via face-to-face (F2F) lectures, which often discouraged HCP with busy schedules from attending such programs. Participants in past programs were often confronted with suddenly scheduling changes at work and had to leave mid-training, while others complained about a lack of time to attend training sessions. Therefore, incorporating technology to provide online training courses has been a rising trend in medical education [12]. This study aims to test the effectiveness of online ESRD CST on the truth-telling confidence and SDM ability of HCP.

## Methods

### Study design

This single-center, single-blind, randomized study employing repeated measures tested the online ESRD CST. The outcomes were the truth-telling confidence and SDM ability of HCP.

### Recruitment

This study was approved by the Institutional Review Board of the study site (No. 201701611B0). Participants were recruited between December 2017 and November 2018 from the nephrology department of a medical center and teaching hospital in northern Taiwan. Inclusion criteria were HCP who were nephrologists or senior nephrology nurses. Nurses were included in this study because they are a communication bridge between physicians and patients and are the best candidate for the decision coach [13, 14]. The principal investigator (PI) introduced the purpose and procedures of this study to potential HCP participants during the monthly nephrology meeting. HCP who were interested in this study were provided with informed consent forms. After HCP completed the informed consent form, participants were randomly allocated to either the intervention group (IG) or the control group (CG) at a 1:1 ratio using computer-generated assignments.

### Intervention

The IG participated in both regular in-service training and the ESRD CST, while the CG was only required to participate in the former. The regular in-service training only included basic communication skills training, while the ESRD CST, which was an advanced form of communication training, consisted of 2 units: truth-telling and SDM for ESRD. Each unit contained two parts: an explanation of the theoretical frameworks for truth-telling and SDM (SHARE & the three-talk model) and video demonstrations of communication with patients with ESRD employing the frameworks mentioned above (Table 1). The ESRD CST lasted approximately 30 minutes and was developed by the PI and the corresponding author (WRT), who are experts in nephrology, medical education, and CST. After developing the preliminary ESRD CST, we asked other experts to confirm its suitability.

**Table 1:** The contents of the regular in-service training and the online ESRD CST course

Course	In-service training	Online CST course
**Course contents**	Basic communication skills training (theoretical lecture)	**1. Truth-telling** • Theoretical framework of SHARE model and video demonstrations of the right (use SHARE model) and wrong methods of disclosure. • Simulation scenario: a middle age female patient who was newly diagnosed with ESRD ^a^. This patient and her husband would be told about the ESRD ^a^ diagnosis by the nephrologist for the first time.
		**2. Shared decision making** • Theoretical framework of Three-talk model and video demonstrations of the right (use Three-talk model) and wrong methods of deliberation. • Simulation scenario: a middle age female patient who was newly diagnosed with ESRD ^a^. The nephrologist discussed treatment options (hemodialysis, peritoneal dialysis, and kidney transplant) with patient and her husband. They decided to receive peritoneal dialysis based on patient’s preference.

^a^ ESRD: end-stage renal disease

### Data collection

Structured questionnaires were used to measure the truth-telling confidence and SDM ability of HCP participants before the intervention (T0), 2 weeks after the intervention (T1), and 4 weeks after the intervention (T2). In addition, we investigated the satisfaction and ESRD CST recommendation intention of the IG participants. To maintain a single-blind design, participants were unaware of the group assignment, and the PI also asked participants not to discuss the intervention they received during the study process to prevent bias, as all participants worked in the same nephrology department. However, trained research assistants provided IG participants with online links to the ESRD CST via mobile device immediately after they had completed the baseline questionnaires (T0); as such, these research assistants had to be aware of the group assignments of the HCP involved. IG participants were required to complete the ESRD CST individually within 2 weeks of receiving the video links on YouTube. A gift certificate was given as an incentive to increase participants’ willingness to complete the study.

### Measures

*Confidence in communication with patients* is a 21-item self-reported scale that was used to test the truth-telling confidence of HCP concerning ESRD patients and their family members [15]. Item responses are rated on a 10-point Likert scale ranging from 1 (not at all) to 10 (extremely). Higher scores indicate greater confidence. This scale exhibits good reliability and validity [16–18]. In this study, Cronbach’s α was .98.

The *Combined Outcome Measure for Risk Communication and Treatment Decision-Making Effectiveness (COMRADE)* is a 20-item self-reported scale, including two subscales, risk communication and confidence in decisions, that aims to measure patients’ thoughts concerning risk communication and confidence concerning treatment decisions. The PI was authorized by the author of the questionnaire (Dr. Edward) to shift the target population to HCP to measure their ability to apply SDM skills. Item responses are rated on a 5-point Likert scale ranging from 1 (strongly disagree) to 5 (strongly agree) [19]. Higher scores indicate that the HCP is more satisfied with risk communication and the decision-making of the HCP is more effective [20–22]. COMRADE has been used in SDM studies concerning patients who had chronic diseases and has demonstrated high reliability (Cronbach’s α = .92 ~ .98) [19, 20, 23]. However, COMRADE has not been used for the HCP population; its internal consistency was tested for the first time in this study (Cronbach’s α = .97).

*Satisfaction with and recommendation intention for ESRD CST* was a single-item indicator that asked IG participants about their satisfaction and intent to recommend the intervention. Item responses are rated on a 5-point Likert scale ranging from 1 (strongly dissatisfied/would not recommend) to 5 (strongly satisfied/would recommend).

### Statistical analyses

IBM SPSS Statistics V24.0 (IBM Corp, Armonk, NY) was used for the analysis; *P* < .05 was considered to be statistically significant; data were analyzed individually. Group differences in baseline data and important outcome variables at each time point were compared by independent t-test and chi-squared test. In addition, to compare group differences in outcome variables over the study period, we used generalized estimating equations (GEEs) to account for within-subject dependency due to repeated measurements and to allow for within-group variation at each time point [24].

To date, no studies have examined the effectiveness of ESRD CST in improving the abilities of HCP concerning truth-telling and SDM with ESRD patients. By relying on the results of past CST studies regarding cancer truth-telling, the PI was aware that F2F CST has a moderate effect size (Cohen’s d = 0.72) [25]. Therefore, the PI decided to use the GEE sample size formula proposed by Prajapati et al. to estimate the required sample size for this study [26]. By setting the power to .80, *p* < .05, and effect size = 0.72, the estimated total sample size was 96 (48 per group). We ultimately enrolled 100 HCP (50 per group) in this study.

## Results

### Participants’ characteristics

**Fig. 1:**
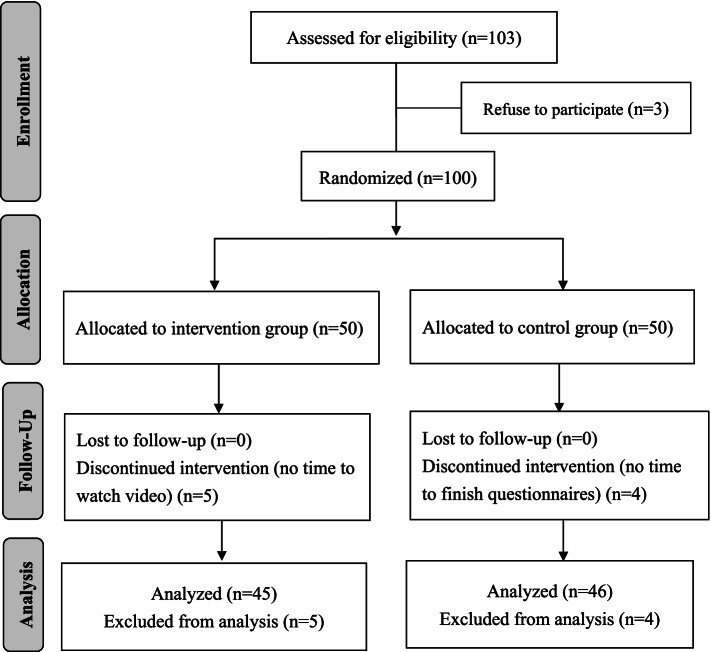
CONSORT flow diagram of participants

Participants were randomized to the IG and the CG. However, five IG participants were unable to complete the ESRD CST in time, and four CG participants did not complete the posttest questionnaire. Therefore, these nine participants were withdrawn from this study (a 9% attrition rate) and excluded from the analysis (Fig. 1). There were no significant differences between participants who were retained or excluded from the study.

The mean age of participants was 41.6 years (SD = 7.6), and most participants were female (80.2%). The largest proportion comprised registered nurses (64.8%) with bachelor’s degrees (47.3%). Half of the participants had prior experience participating in truth-telling (51.6%) or SDM-related courses (49.5%). No significant group differences were found in participants’ characteristics at baseline (Table 2).

**Table 2:** Participants’ characteristics (*N* = 91)

Variables	IG, ***n*** = 45	CG, ***n*** = 46	
	n (%)	n (%)	χ^**2**^ / t (***P***)
**Age (mean ± SD)**	41.7 ± 7.2	41.1 ± 8.2	−0.358 (0.721)
**gender**			
Male	7 (15.6)	11 (23.9)	0.544 (0.461)
Female	38 (84.4)	35 (76.1)	
**Education**			
Associate degree	18 (40.0)	13 (28.3)	1.436 (0.488)
Bachelor’s degree	19 (42.2)	24 (52.2)	
Graduate degree	8 (17.8)	9 (19.6)	
**Years of work**			
4–6 years	3 (6.7)	4 (8.7)	0.685 (0.710)
7–9 years	3 (6.7)	5 (10.9)	
> 10 years	39 (86.7)	37 (80.4)	
**Work position**			
Attending Physician	9 (20.0)	11 (23.9)	0.539 (0.764)
Nurse Practitioner	7 (15.6)	5 (10.9)	
Registered Nurse	29 (64.4)	30 (65.2)	
**Ever taken any communication courses related to truth-telling**			
Yes	24 (53.3)	23 (50.0)	0.012 (0.914)
No	21 (46.7)	23 (50.0)	
**Ever taken any communication courses related to SDM**			
Yes	27 (60.0)	18 (39.1)	3.172 (0.075)
No	18 (40.0)	28 (60.9)	

*IG* intervention group, *CG* control group, *SDM* shared decision making

### Group differences in HCP truth-telling confidence concerning ESRD patients

No significant group differences in truth-telling confidence were observed at any time point, except for T1. The confidence level of the IG was significantly higher than that of the CG at T1 (*t* = 2.83, *P* = .006, Cohen’s d = 0.59) (Table 3). For GEE analysis, a significant group × time interaction was observed only at T1 (*β* = 9.48, *P* = .018) (Table 4).

**Table 3:** Group Differences in Outcome Measure Scores at Each Measurement Time (*N* = 91)

Outcome	Measurement time	IG, ***n*** = 45	CG, ***n*** = 46	t (***P***)
Confidence in communication	T0	150.60 ± 21.86	146.83 ± 24.78	0.770 (0.443)
	T1	165.71 ± 22.15	152.46 ± 22.45	2.833 (**0.006**)
	T2	165.42 ± 21.60	160.48 ± 20.22	1.127 (0.263)
COMRADE—Total	T0	80.06 ± 11.91	76.61 ± 11.89	1.382 (0.171)
	T1	81.73 ± 13.16	79.80 ± 9.16	0.810 (0.420)
	T2	82.40 ± 13.43	80.13 ± 10.47	0.900 (0.370)
COMRADE—Risk communication	T0	41.49 ± 6.68	39.57 ± 6.73	1.368 (0.175)
	T1	42.71 ± 5.79	41.11 ± 5.05	1.408 (0.163)
	T2	42.07 ± 7.26	40.70 ± 7.36	0.894 (0.373)
COMRADE—Confidence in decision	T0	38.57 ± 5.71	37.04 ± 5.75	1.268 (0.208)
	T1	39.02 ± 8.09	38.70 ± 4.66	0.237 (0.814)
	T2	40.33 ± 6.58	39.44 ± 5.26	0.720 (0.473)

T0: before intervention; T1: two weeks after intervention; T2: four weeks after intervention

*IG* intervention group, *CG* control group, *COMRADE* combined outcome measure for risk communication and treatment decision making effectiveness

**Table 4:** Effectiveness of the online CST course on HCP’s truth-telling confidence and SDM ability (*N* = 91)

Model	B	SE	95% CI		***P***
			Lower	Upper	
Confidence in communication					
Group **×** Time ^a^					
IG × T1	9.48	4.02	1.61	17.35	**0.018**
IG × T2	1.17	4.78	−8.20	10.54	0.807
COMRADE—total					
Group **×** Time ^a^					
IG × T1	−1.52	2.08	−5.60	2.56	0.466
IG × T2	−1.18	2.67	−6.41	4.06	0.659
COMRADE—Risk communication					
Group **×** Time ^a^					
IG × T1	−0.32	1.20	−2.67	2.03	0.789
IG × T2	−0.55	1.67	−3.83	2.73	0.741
COMRADE—Confidence in decision					
Group **×** Time ^a^					
IG × T1	−1.20	1.18	−3.51	1.11	0.310
IG × T2	−0.63	1.27	−3.12	1.87	0.624

Reference group = control group; reference time = T0 (baseline assessment)

Adjust: Ever taken any courses related to truth-telling, ever taken any courses related to SDM

*IG* intervention group

^a^ interaction between group and time

### Group differences in HCP SDM ability concerning ESRD patients

No significant group differences were found at any time point in the COMRADE overall (*t* = 0.81 ~ 1.38, *P* = .171 ~ .420) or at any subscale (*t* = 0.24 ~ 1.41, *P* = .163 ~ .814) (Table 3). For GEE analysis, no significant group × time interactions were observed in the overall or any subscale scores at any time point (*β* = − 0.25 ~ − 1.52, *P* = .310 ~ .789) (Table 4).

### Satisfaction and ESRD CST recommendation intention

Over 95.6% of IG participants were satisfied with the ESRD CST, and over 91.1% of participants were willing to recommend participation in ESRD CST to colleagues.

## Discussion

### Principal results

This study aimed to improve the truth-telling confidence and SDM ability of HCP via online ESRD CST. Although only short-term effectiveness in truth-telling confidence between groups was observed, IG participants rated the intervention highly and were willing to recommend participation in ESRD CST to colleagues.

### The effectiveness of ESRD CST on the truth-telling confidence of HCP

This study found that the ESRD CST had a significant short-term effectiveness in enhancing the truth-telling confidence of HCP. Fujimori et al. conducted a study of 30 oncologists to test the effectiveness of a two-day CST program in improving participants’ communication confidence 2 weeks after the CST workshop. Their results showed that IG participants increased their level of confidence significantly more than did CG participants (*F* = 11.2, *P* = .002), which was consistent with the result of this study concerning the short-term (2 weeks) effectiveness of the truth-telling confidence of HCP. However, since Fujimori et al. did not assess the long-term effectiveness of CST in increasing participants’ communication confidence [17], it was difficult to ascertain whether the study found only short-term effectiveness.

In contrast to the short-term effectiveness revealed by this study, two Japanese studies tested the effectiveness of a short (5–6 hours) CST workshop concerning increasing physicians’ and nurses’ truth-telling ability and found that participants’ confidence levels 3 months after the CST workshop were significantly higher than the levels recorded before and immediately after the CST workshop, which demonstrated the long-term effectiveness of CST [16, 18]. However, the CST workshops used in the studies discussed above were conducted through a combination of F2F lectures and role-playing (a simulated form of interaction between HCP and simulated patients intended to implement the expected outcome), which differed from the online CST used in this study.

Even though the ESRD CST showed short-term effectiveness concerning truth-telling confidence, the study design cannot distinguish between the impact of additional CST content and the impact of online delivery. However, our previous studies which delivered CST F2F revealed a moderate to high positive effect size of CST (Cohen’s d = 0.8–0.99) on confidence in communication or perceptions of truth-telling among physicians [25, 27], nurses [25, 28], and interdisciplinary medical staff [25, 27]. Although experts suggested that the online learning method allowed HCP to learn at their own pace [29], and many studies indicate that online courses are just as effective as F2F lectures [30–33], our first attempt to provide CST online found only a moderate effect size on confidence in communication (Cohen’s d = 0.59). Therefore, we believe that the effectiveness may come from additional CST content, not the online delivery. To verify that CST effectiveness is from additional content, we will explore the effectiveness of CST in a forthcoming study targeting the same content (truth-telling and SDM) while controlling for delivery method (online vs. F2F). Additionally, current online learning still limits interaction between the instructor and the learners, which hinders the study effectiveness and overall experience of the learner [34, 35]. Therefore, F2F courses are more rewarding for students than online courses. To mitigate the limitations of online courses, it is recommended to assess the frequency and time spent on learning, as well as to design interactive feedback opportunities to increase the effectiveness of online CST [31, 34, 36, 37].

In addition, the limited training time (30 minutes) might also be a reason for such short-term effectiveness. Scholars have suggested that CST should be followed by posttraining consolidation workshops to maximize effectiveness and to encourage participants to apply what they have learned to daily clinical practice [38–41]. However, this study did not include a posttraining consolidation workshop. Therefore, to improve the effectiveness of online ESRD CST, we suggest that future studies should offer posttraining consolidation workshops to sustain the learning effect and then examine the long-term effectiveness.

### The effectiveness of ESRD CST on the SDM ability of HCP

There were no significant group differences in the COMRADE scores of participants 2 weeks and 4 weeks after the intervention. In contrast to past studies that utilized the COMRADE questionnaire based on patients’ self-reports [20, 42–44], this study was the first to use the COMRADE questionnaire as a self-reported measurement tool for physicians and nurses to assess their SDM abilities. Therefore, the results of this study cannot be directly compared with those of past studies. We also recommend that observation-based assessments, such as observing HCP interactions with patients during truth-telling and SDM processes, be added to future studies to increase the objectivity of the studies.

### Limitations

Several limitations must be considered when interpreting the study results. First, using self-report measures as our main outcome was a major limitation. This approach may entail the risk of self-recall bias. In addition, self-reported measures cannot assess behavioral changes, which may limit the generalizability of this study. Although direct observation in a real-life clinical setting is an objective method for evaluating the effectiveness of the CST concerning behavioral change, this approach may also face certain limitations [40]. A review article found that using behavioral observation as a measurement of real-life encounters had limited effectiveness on physicians’ communication behavior, which was probably due to the Hawthorne effect [45]. Therefore, evaluating learners’ ability via role-playing with simulated patients in a simulated environment is undoubtedly an appropriate method of assessing the highest level of behavioral change outcomes in the context of CST [40, 41]. Second, sampling bias might be another limitation. In this study, 60% of IG participants had attended SDM-related courses prior to the ESRD CST, which meant that they were already familiar with the basic concepts of SDM. Thus, their interest in the ESRD CST may have been diminished, which could in turn have decreased the effectiveness of the intervention on IG participants. Third, the learning experience could have been hindered by the hectic pace of participants’ clinical work. White et al. noted that the more times participants watch videos, the better their study outcomes [33]. However, the clinical work of Taiwanese HCP is very hectic (attending physicians at the study sites conduct approximately 45 outpatient appointments in 3 hours, and nurses are responsible for 8–9 inpatients each). Therefore, after the intervention, IG participants may not have had sufficient remaining time or energy to repeatedly and attentively watch the ESRD CST videos and apply the concepts discussed to their daily clinical work. This situation may have limited the effectiveness of our intervention. Fourth, Taiwanese medical regulations and laws might be another important limitation of this study. A total of 80% of IG participants were nurses, and according to the present law, only physicians are permitted to engage in truth-telling activities and SDM processes. Therefore, there is a possibility that IG nurses were unable to apply what they had learned during the ESRD CST to their daily tasks, which caused the effectiveness of ESRD CST on SDM to be unobservable. However, policy and regulation development takes a long time; offering proper education before regulation and policy have been revised or established is necessary. Therefore, it is essential to provide CST for all HCPs, especially nurses, to develop important communication skills (e.g., truth-telling and SDM) in collaboration with physicians. This education may also accelerate policy and regulation development.

### Clinical implications

This study did not verify the effectiveness of CST on SDM ability among HCPs. Still, because present law in Taiwan does not prevent HCPs from exploring patients’ concerns about and preferences for treatments, all HCPs need SDM education. As a guide for SDM CST, we recommend both the three-talk model and, especially with nurse participants, the Ottawa Decision Support Framework (ODSF). The ODSF emphasizes team effort and recommends a decision coach during the SDM deliberation process [13, 14]. Nurses are the best candidates for decision coaches [13, 14]. On the other hand, although this study was limited and only found a short-term moderate effect in enhancing truth-telling confidence, online curricula is on the rise in medical education due to the COVID-19 pandemic, and further research is needed. Therefore, we suggest that future medical education researchers should consider the limitations of online courses and use this preliminary result as a reference for effective online course design.

## Conclusions

The test of this ESRD CST led to a merely preliminary result. ESRD CST enhanced short-term truth-telling confidence, though it is unclear whether this was due to CST content or the online learning method. Therefore, future studies verifying the effectiveness of online CST must carefully design interactive modules, control for delivery method, and incorporate both subjective and objective evaluations.

## Supplementary Information

**Additional file 1.** IRB Approval.

## Data Availability

The datasets generated and analyzed during the current study are not publicly available due to ethical consideration but are available from the corresponding author on reasonable request.

## Abbreviations

ESRD: End-stage renal disease
SDM: Shared decision-making
CST: Communication skills training
F2F: Face-to-face
PI: Principal investigator
IG: Intervention group
CG: Control group
COMRADE: Combined Outcome Measure for Risk Communication and Treatment Decision-Making Effectiveness
GEE: Generalized estimating equations
