# Supplementary material for: Effectiveness of end-stage renal disease communication skills training for healthcare personnel: a single-center, single-blind, randomized study
Source: BMC Med Educ. 2022 May 23;22:397. doi: 10.1186/s12909-022-03458-9 (PMC9125352; doi:10.1186/s12909-022-03458-9)
Supplement: Supplementary file 1 — Additional file 1. IRB Approval. [file 12909_2022_3458_MOESM1_ESM.pdf]

Chang Gung Medical Foundation
Institutional Review Board
199, TUNG HWA NORTH ROAD,
TAIPEI, TAIWAN, 10507
REPUBLIC OF CHINA
Tel: (03) 3196200
Fax: (03) 3494549

Date 2018/01/02

Protocol Title: Holistic Care Quality Improvement Project: Taking Patients with End Stage Renal Disease for Example
IRB No.: 201701611B0C501
Principal Investigator(s): JI-TSENG FANG
Co-Investigator(s): LEE, CHIEN-HUNG,KAO, CHEN-YI,WU, I-WEN,Chung-Chih Lin,Woung-Ru, Tang,YA-CHUNG TIAN
Executing Institution: Taipei,Keelung,Linkou,Taoyuan
Duration of Approval: From 2018/01/02 TO 2018/11/30
Approved Protocol: 2017/10/27 Version2
Approved Informed Consent: 2017/11/08 Version3; 2017/11/08 Version3
Date of Approval: 2018/01/02

※The research will expire on 2018/11/30. Please submit the final report to the IRB for review within three months after the expiration of the trial research. For extension of the trial period, please submit the amendment and a continuing report two months before the expiration.

The IRB is organized and operates in accordance with Good Clinical Practice and the applicable laws and regulations.

T. T. Hsieh

Tsang-Tang Hsieh,MD
Chairman
Institutional Review Board
Chang Gung Medical Foundation
